# Sex-dependent effects of forced exercise in the body composition of adolescent rats

**DOI:** 10.1038/s41598-021-89584-8

**Published:** 2021-05-12

**Authors:** Y. Kutsenko, A. Barreda, A. Toval, D. Garrigos, M. Martínez-Morga, B. Ribeiro Do Couto, J. L. Ferran

**Affiliations:** 1 Department of Human Anatomy and Psychobiology, Faculty of Medicine, University of Murcia, Murcia, Spain; 2 Institute of Biomedical Research of Murcia – IMIB, Virgen de La Arrixaca University Hospital, Murcia, Spain; 3 Faculty of Psychology, University of Murcia, Murcia, Spain

**Keywords:** Weight management, Computed tomography, Whole body imaging, Obesity

## Abstract

Determining the body composition during adolescence can predict diseases such as obesity, diabetes, and metabolic syndromes later in life; and physical activity became an effective way to restore changes in body composition. However, current available literature assessing the body composition before, during and after adolescence in female and male rodents by in vivo techniques is scarce. Thus, by using computerized tomography, we aimed to define the baseline of the weight and body composition during the adolescence and young adulthood of female and male Sprague–Dawley rats (on P30, P60 and P90) under standard diet. Then, we determined the effect of 18 days of forced exercise on the body weight and composition during the early adolescence (P27-45). The highest percentual increments in weight, body volume and relative adipose contents occurred during the female and male adolescence. Forced running during the early adolescence decreased weight, body volume and relative adipose delta and increment values in males only. The adolescence of rats is a period of drastic body composition changes, where exercise interventions have sex-dependent effects. These results support a model that could open new research windows in the field of adolescent obesity.

## Introduction

Adolescence is the transition between childhood and adulthood, a period of vulnerability to develop mental illnesses and marked by maturational changes of extensive brain regions^1–5^. Also, it is characterized by a risk to develop obesity due to eating habits, sexual maturation and decline of physical activity^6,7^. According to critical neurobiological changes observed in the neural circuits of the prefrontal cortex, the adolescent period of rats can be defined between P30 and P60^1,2,5^. Adolescence is a key period to determine the body composition since it can predict diabetes, obesity, osteoporosis, and cardiovascular diseases later in life^8^. Human reports indicate that an increased adipose tissue content that causes overweight or obesity during adolescence, determines a higher risk of obesity in the adulthood^6,9^. However, changes in body composition due to physical activity during adolescence in rodent models have been little explored.

Physical activity is an effective way to prevent or restore changes in body composition in diseases such as obesity, diabetes, or metabolic syndrome, since it helps to improve the energy balance^10^. Physical activity also stimulates the central nervous system (CNS), improving cognitive function, cardiovascular system, or modifying key metabolic responses^11–23^. Studies in humans indicate that low levels of physical activity during adolescence appear as one of the main causal factors for overweight and obesity, and increase the risk of being obese during adult life^6^. The effects of physical exercise on the body composition in rats were mainly determined during adult periods of life, with few studies in adolescence^24–28^. In addition, only a low number of studies in rodents used reliable and accurate modern techniques to determine the effects of exercise on body composition^29,30^.

The first studies in rodents that determined the body composition included ex-vivo dissection^26,31–33^ and in-vivo bioimpedance or gas uptake techniques^34–36^. Unfortunately, these methods do not allow to assess minimal changes or analyze the whole-body composition, specific anatomical locations or perform longitudinal studies. Among the recent imaging techniques, dual-energy X-ray absorptiometry (DXA) could be a reliable method to regionalize body composition^37^. On the other hand, magnetic resonance (MR) allows to accurately identify hard and soft tissues and distinguish brown (BAT), subcutaneous (SAT) and visceral (VAT) adipose tissues^38–40^. However, only high-resolution MR techniques allow to regionalize different tissues of the body. This is also achieved with similar accuracy by using positron emission tomography (PET)^41,42^. Nonetheless, MR and PET require costly resources that are out of reach for many laboratories. One increasingly used method to analyze the body composition in animal studies is the computerized tomography (CT)^43–46^. CT shows a high correlation with MR imaging regarding adipose and lean tissue volumes when no post-filtering processes are used during the image analysis^47^. In addition, CT techniques are not as costly as MR and PET, while providing an accurate measurement of body composition and its distribution. Recent studies highlighted the importance of individually analyzing subcutaneous and visceral adipose tissue due to their differentiated metabolic relationships in a wide range of diseases, including cardiovascular diseases, cancer, and metabolic syndromes^48,49^. In this sense, computerized tomography (CT) imaging is revealed as an appropriate method for body composition studies in longitudinal studies^50,51^.

In this study, by using computerized tomography, we first aimed to define the baseline of the weight and body composition during adolescence (P30-60) and young adulthood (P60-90) of female and male Sprague–Dawley rats under standard laboratory chow diet. Then, during the early adolescence (P27-44), we determined the effect of a forced wheel training program on the weight and body composition. The forced running wheel modality allows to accurately determine the effects of physical activity during adolescence since it ensures the exposure to similar loads of exercise (volume, speed and frequency) and can avoid unspecific stress responses in rodents^52–54^. Our study derived in a detailed assessment of the body composition by using CT. According to our baseline, the highest percentual increments in weight, body volume and relative adipose contents mainly occur during the adolescence when compared with the young adulthood. Finally, we observed that an aerobic physical activity program during the early adolescence has sex-dependent effects on the body composition of Sprague–Dawley rats. These results highlight the relevance of including both sexes in the animal studies and suggest that future studies revealing the differential mechanisms of exercise between females and males could open new research windows about obesity in the adolescence.

## Material and methods

The study was carried out in compliance with the ARRIVE guidelines^55^. Experimental procedures and animal care were approved by the ethic committee of the University of Murcia (Authorization Number: REGA ES300305440012) and therefore conducted according to the guidelines stated by the Spanish (1201/2005) and the European Union (86/609/EEC) regulations.

### Animals and housing

36 Sprague–Dawley rats provided by the Animal Facilities of the University of Murcia were used in the experiment. The animals arrived at the experimental facilities on postnatal day 20 (P20). Female baseline group (FB; n = 6; litter A) and male baseline group (MB; n = 6; litter B) were housed in separate cages (3 rats per cage) connected to individual ventilation racks. Female training group (FT; n = 6; litter C and litter D), female control group (FC; n = 6; litter C and litter D) were weight-matched and composed by 3 animals from litter C and 3 animals from litter D, and housed in a separate room under the same conditions. Male training group (MT; n = 6; litter E) and male control group (MC; n = 6; litter E) were weight-matched and housed in a separate room under the same conditions. Standard size cages, of 50 × 35 × 35 cm with a 2–3 cm dry cork shavings layer, were replaced every 4–5 days. A 12:12 h dark–light cycle was set, being zeitgeber (ZT) 0 when the lights turned on (rat’s passive phase), and ZT12 when the lights turned off (rat’s active phase). All the experiments were performed during the rats’ active phase (dark cycle) under a source of dim orange light (600 nm), at ZT14 and/or ZT20 to minimize circadian disruptions. The rooms were maintained at 22–25 °C temperature with a relative humidity of 45–60%. Standard chow diet (ENVIGO, diet 2014) and filtered water were available ad libitum.

### Forced wheel protocol

**Figure 1:**
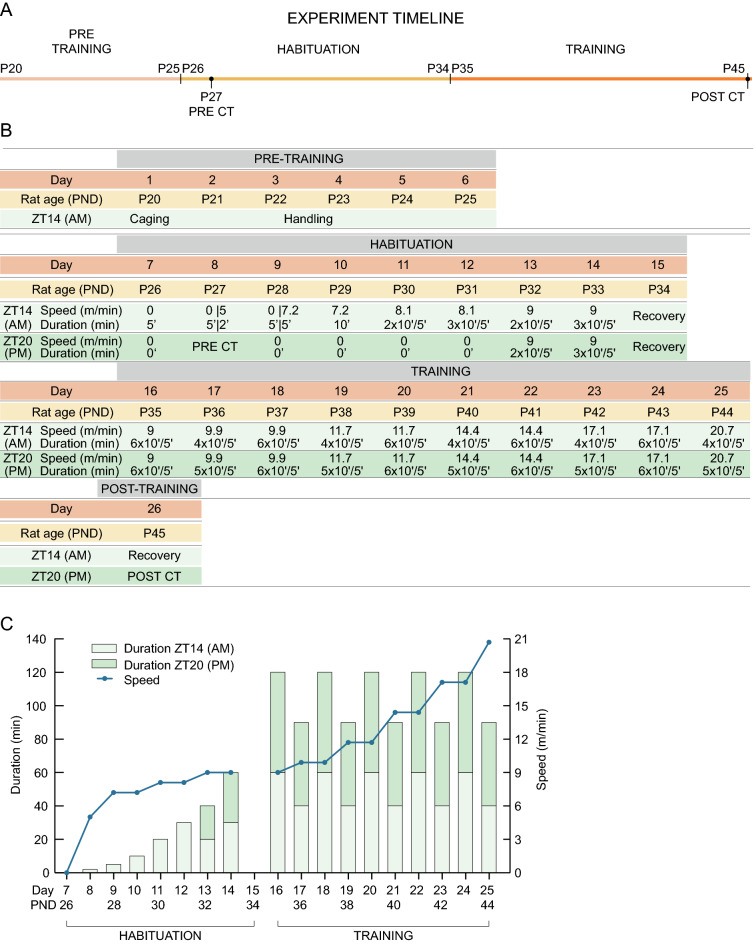
Forced wheel exercise schedule and protocol. (**A**) Experiment timeline. (**B**) Forced exercise schedule and protocol. The training program was divided in 4 steps, namely Pre-training, Habituation, Training and Post-training. The duration of the exercise was split in bouts of 10 min, with a resting period of 5 min between bouts. (**C**) Representation of the duration (bars) and speed (line) of the training program.

The training was designed and supervised in collaboration with graduates in physical activity and sport science, certificated and with permission to work with experimental animals. The handling procedure consisted of picking up one rat at a time and petting it for 2 min. From postnatal day 26, female and male animals of the experimental groups were exposed to an 8-days exercise habituation protocol in a forced running wheel system (Campden Instruments, 80805A) as described previously (Fig. 1)^52^. The training program consisted of two sessions per day, on ZT14 and ZT20. The active period of SD rats occurs during the darkness (ZT12-ZT23). ZT14 session was defined as morning (AM) session, since it occurs in the first half of the active phase. ZT20 was defined as afternoon (PM) session, since it occurs in the second half of the active phase. On each session (AM and PM), the rats were weighted (Gram S3R-2KD) before entering the wheel system. The sessions consisted of 10-min bouts of continuous running, with 5 min of resting between bouts. The speed and/or total duration increased every day (Fig. 1). The rotation speed of the wheels was established according to the response of the rats in preliminary experiments.

### Computerized tomography

On postnatal day 30, 60 and 90 of the FB and MB groups and postnatal day 27 and 45 of the FT, FC, MT and MC groups, the rats were screened using an Albira trimodal preclinical-scanner and software (Bruker®, Billerica, MA, USA). The rats were anesthetized (MSS isoflurane Keyed Filler Cagemount, MSSVAP03) through inhalation of isoflurane (ZOETIS, IsoFlo) at 5% in a 30 × 15 × 15 cm chamber until the absence of voluntary movement. After the anesthesia induction, the rats were weighted (Gram S3R-2KD) and prepared for image acquisition. Then, the isoflurane flow was reduced to 2.5–3% and mixed with a 1.6–1.8 L/min of oxygen flow (DeVilbiss Healthcare, 5 L Oxygen Concentrator) until the end of the scanning. The image scanning process took between 25 and 40 min per rat. The x-ray intensity parameters were set to 45 kV and 0.4 mA. P27 and P30 rats were exposed to approximately 893.9 SDE mSv (or 89.4 rem) and 550 DDE mSv (or 55 rem), while P45, P60 and P90 rats received approximately 1,430.24 SDE mSv (or 143 rem) and 880 DDE mSv (or 88 rem). Three sets of 600 projections of 0.05 mm voxel size were obtained using a digital flat panel detector of 2400 × 2400 pixels and a 70 × 70 mm field of view. Once the acquisition was complete, the rat recovered from anesthesia through pure oxygen inhalation and a source of heat (Cibertec, Rtc-0 Thermo Controller Blanket System). In addition to the animal imaging, one more acquisition was performed without the animals, to separate the background noise in the image analysis. The images were spatially reconstructed through the filtered back projection algorithm (Albira reconstruction software). The researchers responsible for the image acquisition process were blind to the groups.

### Image analysis

**Figure 2:**
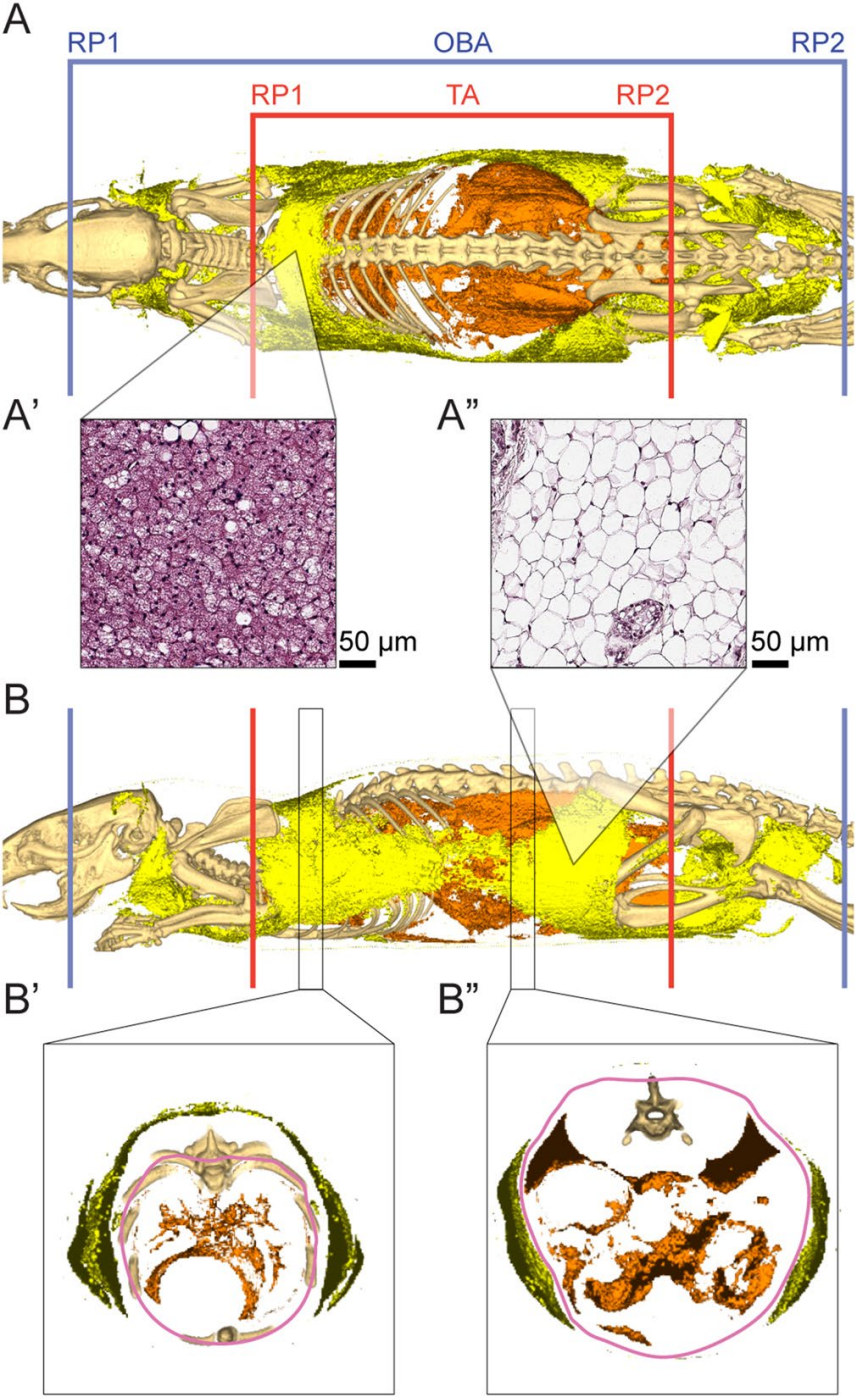
Reference points of the image analysis. (**A**) Dorsal view of a scanned rat. The thoracoabdominal region was comprised by the reference points TA RP1 and TA RP2 (red lines). The OBA region is delimited between the posterior boundary of the olfactory bulb and the anus, by the reference points OBA RP1 and OBA RP2 (blue lines) The VAT is represented in orange, and the SAT in yellow. (**A’** and **A”**) Histological view of BAT (**A’**) and WAT (**A”**). (**B**) Lateral view of a scanned rat. (**B’** and **B”**) Transversal view of two 3.7 mm-sections from B. The manual contour (pink) was drawn inside the muscle/bone plane of the thoracoabdominal wall.

The 3D image segmentation was performed using the pMod version 3.5. With the software’s reduction tool, the image resolution was halved to substantially improve the processing time. The full volume of the rat was calculated by using the “isocontour” function of the software with the following parameters: algorithm ‘3D’; search mode ‘hot’; inner holes ‘no’; multi regions ‘no’; mode of searching ‘automatic’; absolute threshold ‘value’; threshold ‘-450’. The thoracoabdominal (TA) region was analyzed and defined as the contents from the intervertebral space C7-T1 (cervical and thoracic) to the intervertebral space between S2–S3 (sacral). A broader region was analyzed and defined between the posterior boundary of the olfactory bulb and the anus (olfactory bulb-anus; OBA region) (Fig. 2A,B). The method did not permit to differentiate between white adipose tissue (WAT) or brown adipose tissue (BAT) (Fig. 2A’,A”), and therefore we only considered the visceral (VAT) and subcutaneous (SAT) adipose tissue. To split the VAT from the SAT, a contour was drawn manually with the software. The VAT region was defined as the contents comprised by the mentioned contour, inside of the muscle/bone plane of the thoracoabdominal wall of the TA region (Fig. 2B,B”). The adipose tissue outside the described limits was defined as SAT. To ensure the accuracy of the compartment separation, 2 contours were drawn by 3 different researchers, and then compared for differences to reach an agreement. Due to the limitations of the image quality, separating VAT from SAT was not possible in the OBA region. Therefore, the analysis of the OBA region does not include the separation between SAT and VAT. The sum of VAT and SAT was defined as the total adipose tissue (AT) content. Based on calibration standards that were validated previously in data from humans and rodents^43,56^, using the “segmentation” functionality of the software, three ranges of signal were obtained and considered in terms of adipose (− 200 to − 31 HU), lean (− 30 HU to 189 HU; note that lean tissue includes mainly muscle, but also includes the parenchyma and stroma of organs like kidneys, liver or intestines) or bone tissue (≥ 190 HU). The raw segmentation of the image was used in all the calculations. Therefore, no post-processing was used after the segmentation. Body composition contents were reported as the percentage of the corresponding body volume. The 3D images (Sup. 2; Sup. 5; Sup. 6) were created by loading the CT raw images in VolView 3.4 (Kitware), adjusting the radiodensity parameters until only the bone is visible, and merging it (by clicking on Plugins > Utilities > Merge volumes) with the segmented SAT and VAT volumes, prior to choosing a different color for every tissue. The 3D videos (Vids. 1–14) were created by using the VolView tools (Review > Movie > Create). The researchers responsible for the image analysis were blind to the groups.

### Statistical analysis

The SPSS v25 was used to perform all the statistical analyses. First, the normality of the data was tested by using the Shapiro–Wilk test. The outliers were statistically identified by assessing the boxplots of the normality tests. Only the outliers that resulted in a change of the statistical outcome were removed. A mixed model of 2-way ANOVA (or split-plot ANOVA) was used to analyze the main effects and interactions of within-subjects and between-subjects variables regarding relative and absolute values (see results). In case of sphericity violation, Huynh-Feld test was used to establish significance. Between-subjects comparisons were then followed-up with Fisher’s LSD post-hoc adjustment. Paired T-tests were used for all within-subjects paired comparisons. Delta and increment comparisons that were between-subjects (see results) were analyzed using U-Mann Whitney or T-student, in accordance to the normality of the data. Significance level was established at *p* < 0.05.

## Results

**Figure 3:**
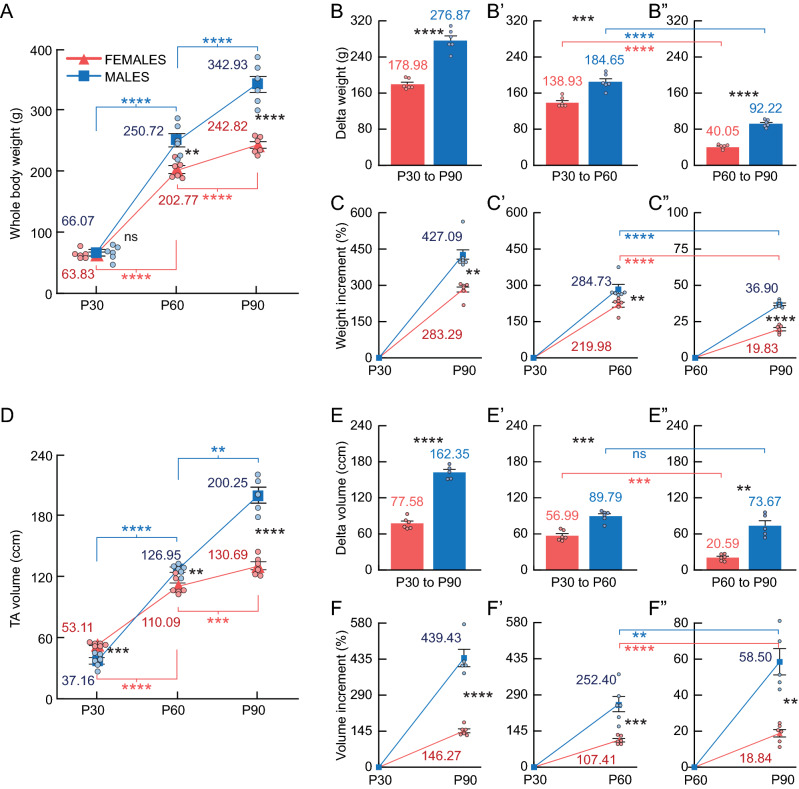
Body weight and thoracoabdominal (TA) volume of female (red) and male (blue) rats. (**A**) Raw whole-body weight (g) on P30, P60 and P90. The split-plot ANOVA revealed an interaction between age and sex (F_1.3,20_ = 89.70, *p* < .01), with a main effect of age (F_1.3,13.2_ = 1978.56, *p* < .01) and sex (F_1,10_ = 25.79, *p* < .01) in body weight. (**B**, **B’** and **B”**) Delta weight (g) from P30 to P90, P30 to P60 and P60 to P90. (**C**, **C’** and **C”**) Percentual increment of body weight (%) from P30 to P90, P30 to P60 and P60 to P90. (**D**) TA volume (ccm). The split-plot ANOVA revealed an interaction between age and sex (F_2,20_ = 104.48, *p* < .01), with a main effect of age (F_2,20_ = 820.84, p < .01) and sex (F_1,10_ = 40.47, *p* < .01) in TA volume. (**E**, **E’** and **E”**) Delta TA volume (ccm). (**F**, **F’** and **F”**) Percentual increment of TA volume (%). **Statistics**: Between-subjects effects were followed with Fisher’s LSD post-hoc. Paired T-test was used for within-subjects comparisons. Unpaired T-test was used in delta and increment between-subjects comparisons. Black stars (*): female versus male. Blue symbols: within-male comparisons. Red symbols: within-female comparisons. Values represented as mean and SEM. Significance levels: *p < .05, **p < .01, ***p < .001, ****p < .0001, ns: *p* > .05.

**Figure 4:**
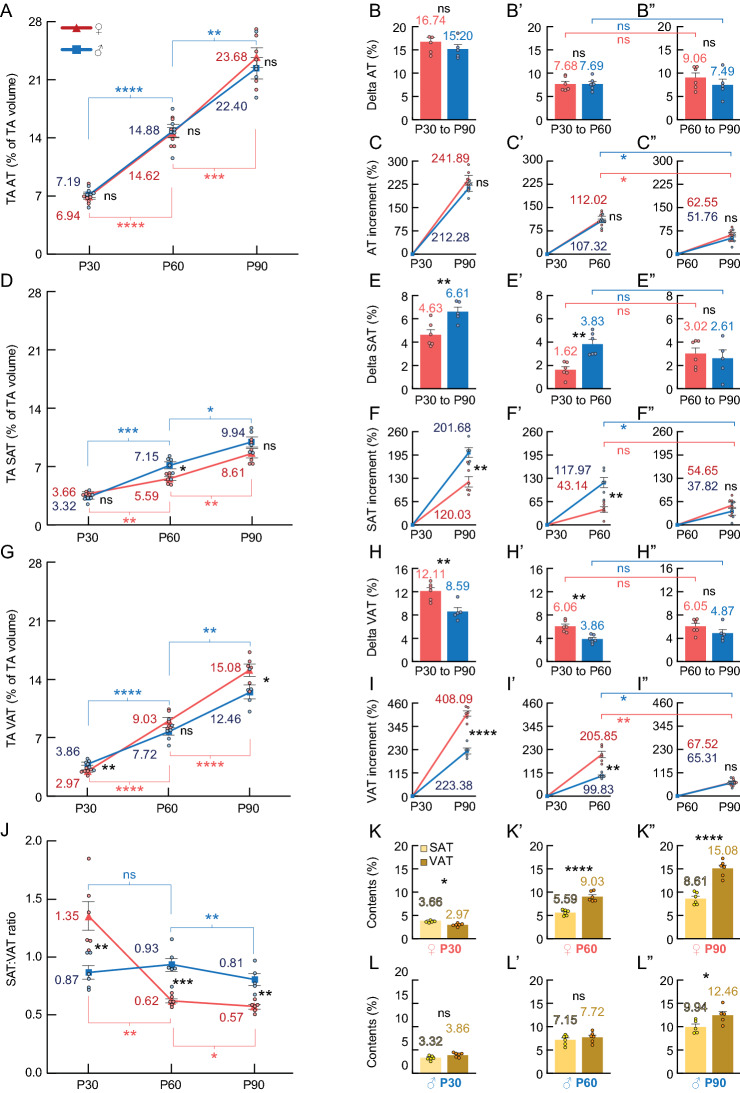
Female (red) and male (blue) comparisons of TA adipose contents. (**A**) Relative adipose contents (AT%). The split-plot ANOVA revealed a main effect of age in TA AT% (F_2,20_ = 365.11, *p* < .01). (**B**, **B’** and **B”**) Delta AT%. (**C**, **C’** and **C”**) Percentual increment (%) of AT%. (**D**) Relative subcutaneous adipose contents (SAT%). The split-plot ANOVA revealed an interaction between age and sex in TA SAT% (F_2,20_ = 5.93, *p* = .010), with a main effect of age (F_2,20_ = 169.77, *p* < .01) and sex (F_2,20_ = 5.93, *p* = .010). (**E**, **E’** and **E”**) Delta SAT%. (**F**, **F’** and **F”**) Percentual increment of SAT%. **G)** VAT%. The split-plot ANOVA revealed an interaction between age and sex in TA VAT% (F_2,14_ = 12.08, *p* < .01), with a main effect of age (F_2,20_ = 447.98, *p* < .01). (**H**, **H’** and **H”**) Delta VAT%. (**I**, **I’** and **I”**) Percentual increment of VAT%. (**J**) SAT%:VAT% ratio. The split-plot ANOVA revealed an interaction between age and sex in the TA SAT%:VAT% ratio (F_1.3,12.5_ = 33.39, *p* < .01), with a main effect of age (F_1.25,12.52_ = 37.91, *p* < .01). (**K–K”**) SAT% (yellow) versus VAT% (brown) comparisons of female rats (paired T-test). (**L–L”)** SAT% versus VAT% comparisons of male rats (paired T-test). **Statistics**: Between-subjects effects were followed with Fisher’s LSD post-hoc. Paired T-test was used for within-subjects comparisons. Unpaired T-test was used in delta and increment between-subjects comparisons. Black stars (*): female versus male (or SAT% vs. VAT% in K–K” and L–L”). Blue symbols: within-male comparisons. Red symbols: within-female comparisons. Values represented as mean and SEM. Significance levels: *p < .05, **p < .01, ***p < .001, ****p < .0001, ns: *p* > .05.

**Figure 5:**
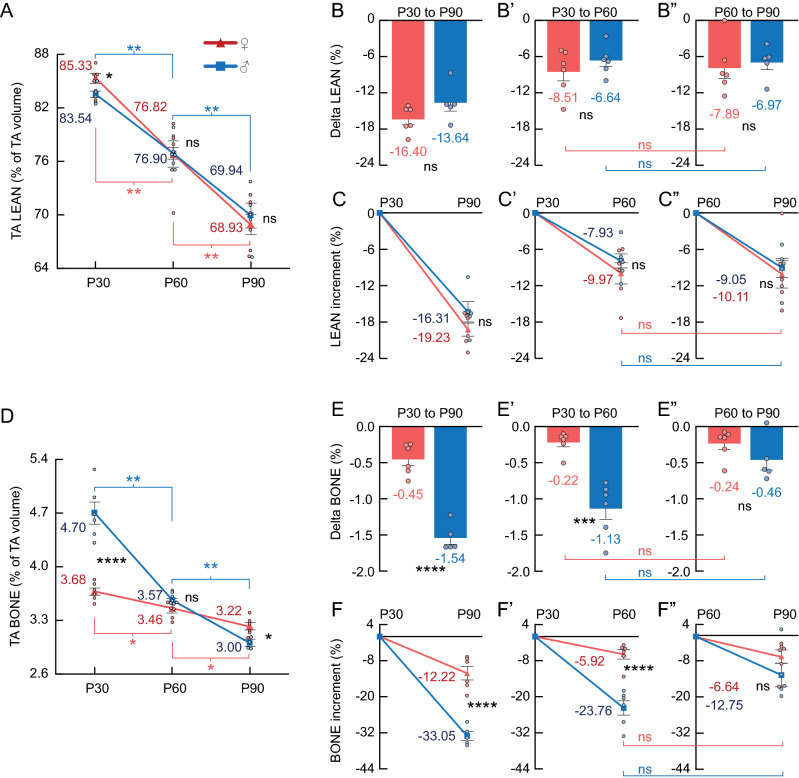
Female (red) and male (blue) comparisons of TA LEAN% and TA BONE%. (**A**) Relative lean contents (LEAN%). The split-plot ANOVA revealed a main effect of age in TA LEAN% (F_2,20_ = 142.98, *p* < .01). (**D**) Relative bone contents (BONE%). The split-plot ANOVA revealed an interaction between age and sex in BONE% (F_2,14_ = 40.45, *p* < .01), with main effects of age (F_2,20_ = 117.51, *p* < .01) and sex (F_1,10_ = 24.35, *p* < .01). (**B–B”** and **E-E”**) Delta LEAN% and delta BONE% (respectively). (**C–C”** and **F-F”**) LEAN% and BONE% (respectively) percentual increment (%). **Statistics**: Between-subjects effects were followed with Fisher’s LSD post-hoc. Paired T-test was used for within-subjects comparisons. Unpaired T-test was used in delta and increment between-subjects comparisons. Black stars (*): female versus male. Blue symbols: within-male comparisons. Red symbols: within-female comparisons. Values represented as mean and SEM. Significance levels: *p < .05, **p < .01, ***p < .001, ****p < .0001, ns: *p* > .05.

**Figure 6:**
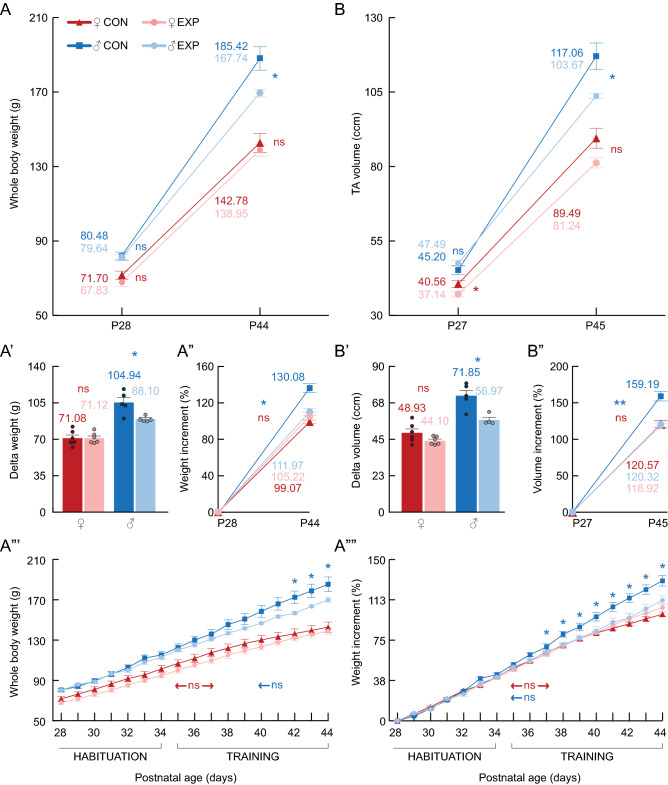
Body weight and TA volume of control and experimental rats. (**A**) Whole-body weight (g). In males, the split-plot ANOVA revealed an interaction between age and treatment in body-weight (F_1,9_ = 7.46, *p* = .023). Females showed no interaction (F_1,10_ < 0.001, *p* = 0.993). (**A’**) Delta body weight. (**A”**) Percentual increment of body weight. (**A”’**) Daily BW (g). (**A””**) Daily relative BW percentual increment (%). (**B**) TA volume (ccm). In males, the split-plot ANOVA revealed an interaction between age and treatment in TA volume (F_1,9_ = 26.70, *p* < .01). Females showed no interaction (F_1,10_ = 3.26, *p* = 0.101). (**B’**) Delta TA volume. (**B”**) Percentual increment of body weight (%). **Statistics**: Between-subjects effects were followed with Fisher’s LSD post-hoc. Paired T-test was used for within-subjects comparisons. Unpaired T-test was used in delta and increment between-subjects comparisons. Blue stars (*): male control versus trained. Red stars (*): female control versus trained. Values represented as mean and SEM. Significance levels: *p < .05, **p < .01, ***p < .001, ****p < .0001, ns: *p* > .05.

**Figure 7:**
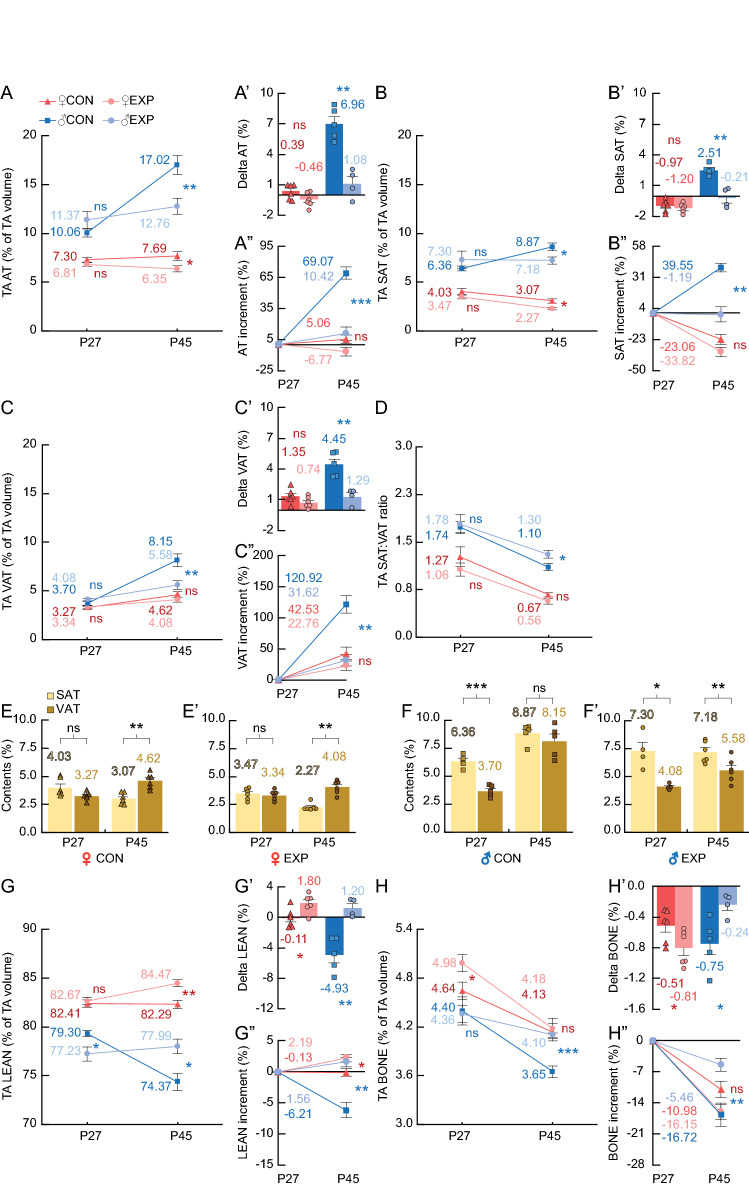
TA adipose (AT%, SAT%, VAT%), LEAN% and BONE% contents of control and trained rats before (P27) and after (P45) the training program. (**A**) Relative adipose contents (AT%). The split-plot ANOVA revealed a TA AT% interaction between age and treatment in males (F_1,9_ = 54.49, *p* < .01), but not females (F_1,10_ = 4.26, *p* = 0.066). (**A’**) Delta AT%. (**A”**) AT% percentual increment. (**B**) Relative subcutaneous adipose contents (SAT%). The split-plot ANOVA revealed a TA SAT% interaction between age and treatment in males (F_1,9_ = 48.15, *p* < .01), but not females (F_1,10_ = 0.59, *p* = 0.461). (**B’**) Delta SAT%. (**B”**) SAT% percentual increment (%). (**C**) Relative visceral adipose contents (VAT%). The split-plot ANOVA revealed a TA VAT% interaction between age and treatment in males (F_1,9_ = 34.07, *p* < .01), but not females (F_1,10_ = 2.87, *p* = 0.121). (**C’**) Delta VAT%. **C”)** VAT% percentual increment (%). **D)** SAT%:VAT% ratio. The split-plot ANOVA revealed no interactions in males (F_1,9_ = 2.25, *p* = 0.168), or females (F_1,10_ = 0.39, *p* = 0.546). (**E–E’**) SAT% (yellow) versus VAT% (brown) comparisons of female control (**E**) and experimental (**E’**) rats. (**F–F’**) SAT% versus VAT% comparisons of male control (**F**) and experimental (**F’**) rats. (**G**) Relative lean contents (LEAN%). The split-plot ANOVA revealed a TA LEAN% interaction between age and treatment in females (F_1,10_ = 7.07, *p* = .024) and males (F_1,9_ = 38.02, *p* < .01). (**G’**) Delta LEAN%. (**G”**) LEAN% percentual increment (%). (**H**) Relative bone contents (BONE%). The split-plot ANOVA revealed a TA BONE% interaction between age and treatment in females (F_1,10_ = 5.46, p = .042) and males (F_1,9_ = 12.80, *p* < .01). (**H’**) Delta BONE%. (**H”**) BONE% percentual increment (%). **Statistics**: Between-subjects effects were followed with Fisher’s LSD post-hoc. Paired T-test was used for within-subjects comparisons. Unpaired T-test was used in delta and increment between-subjects comparisons. Black stars (*) indicate the *p* value level of SAT% versus VAT% in (**E**–**F’**). Blue stars (*): male control versus trained. Red stars (*): female control versus trained. Values represented as mean and SEM. Significance levels: *p < .05, **p < .01, ***p < .001, ****p < .0001, ns: *p* > .05.

A group of female and male SD rats was analyzed on P30, P60 and P90 to define a baseline of body weight and body composition. These key ages defined two life stages, namely adolescence (P30-60) and young adulthood (P60-90). Next, we determined the effect of a forced wheel training during the early adolescence period of female and male rats. In the results below, we show the absolute values of the whole-body weight and the TA or OBA volume of the rats. Then, we normalized the volume of the rats’ adipose, lean and bone volumes, obtained by CT, to the volume of the TA region (Figs. 3, 4, 5, 6, 7) or OBA region (Sup. 1; Sup. 3; Sup. 4) and expressed it as a percentage (relative content). The delta values were reported as the difference between two age points (e.g. P30 to P90 difference = P90 value – P30 value). The percentual increment values were reported as the percentage difference between two age points (e.g. P30–P60 increment = P60 value * 100/P30 value – 100).

### Baseline female and male SD rats experimented the highest increment in weight and volume during adolescence

Baseline female and male rats showed similar weights on postnatal day 30 (Fig. 3A), but females showed higher thoracoabdominal (TA) volume (Fig. 3D). Nonetheless, P60 males presented higher weight and TA volume, being this difference more pronounced on P90 (Fig. 3A,D). Between P30 and P90, the highest delta and increment values were observed in male weight and TA volume (Fig. 3B,C,E,F). Furthermore, males showed higher delta and increment values of weight than females during adolescence (P30–60), and young adulthood (P60–90) (Fig. 3B’,B”,C’,C”). Similar differences were observed regarding the TA volume (Fig. 3E’,E”,F’,F”). To determine the life stage where the most pronounced changes occurred, the percentual increments obtained in the adolescence and young adulthood were compared. Both females and males showed the highest increment of weight and volume during adolescence (P30–60) (Fig. 3C’–C”,F’–F”). Note that the delta values of TA volume of males during adolescence (P30–60) and young adulthood (P60–90) were similar (Fig. 3E’–E”) (see discussion). The analysis of the OBA region revealed mostly similar differences to the TA (Sup. 1B’–B”).

### Baseline female and male SD rats experimented the highest increment in the adipose content during adolescence

In the thoracoabdominal region, the relative adipose (AT%) content -or body fat- increased with age without differences between females and males (Fig. 4A; Sup. 2; Vids. 1–6). No differences were found between females and males regarding the AT% delta and increment values from P30 to P90, adolescence (P30–60) or early adulthood (P60–90) (Fig. 4B–C”). Comparing adolescence (P30–60) with young adulthood (P60-90) the AT% increment was higher during adolescence in both females and males (Fig. 4C’,C”). Note that the delta AT% values in the adolescence and young adulthood were similar (Fig. 4B’,B”) (see discussion). The analysis of the OBA region revealed similar differences to the TA region, except that no difference was observed between adolescence and young adulthood OBA AT% in females (Sup. 3A–C”).

In relation to the SAT%, it increased with age in both females and males (Fig. 4D; Sup. 2A,D,G,J,M,P). In both delta and increment values, males showed higher SAT% than females from P30 to P90 (Fig. 4E,F), specifically in the adolescence (P30–60) (Fig. 4E’,F’), but not in the young adulthood (P60–90) (Fig. 4E”,F”). Comparing adolescence (P30–60) with young adulthood (P60–90), males but not females showed higher SAT% increment during the adolescence (Fig. 4F’–F”).

Regarding the VAT%, it increased with age in both females and males (Fig. 4G). In both delta and increment values, females showed higher VAT% than males from P30 to P90 (Fig. 4H,I), specifically in the adolescence (P30–60) (Fig. 4H’,I’), but not in the young adulthood (P60–90) (Fig. 4H”,I”). Comparing adolescence (P30–60) with young adulthood (P60–90), the VAT% increment in females and males was higher during adolescence (Fig. 4I’,I”). Note that the delta VAT% values of the adolescence and young adulthood were similar in both females and males (Fig. 4H’,H”) (see discussion).

In relation to the SAT%:VAT% ratio, females showed a higher ratio on P30, but lower than males on P60 and P90 (Fig. 4J). Finally, females showed lower VAT% than SAT% on P30, but higher VAT% than SAT% on P60 and P90 (Fig. 4K–K”). On the other hand, males showed higher VAT% than SAT% on P90 only (Fig. 4L–L”).

### Similar increments between adolescence and young adulthood were observed in the LEAN% and BONE%

In the TA region, the LEAN% decreased with age in both males and females (Fig. 5A). No differences were found between sexes or between life stages regarding the LEAN% delta or increment values (Fig. 5B–C”). In contrast to the TA region, the LEAN% delta and increment values in the OBA region were lower in females than males from P30 to P90, specifically during adolescence (P30–60), but not during young adulthood (P60–90) (Sup. 4B–C”).

The BONE% decreased with age in both males and females (Fig. 5D). The BONE% delta and increment values were lower in males than females from P30 to P90, specifically during adolescence (P30–P60), but not during young adulthood (P60–P90) (Fig. 5E–F”). No differences were found between life stages regarding the BONE% delta or increment values (Fig. 5E–F”). Regarding the OBA region, similar BONE% results to the TA region were observed (Sup. 4D–F”).

### AM and PM forced running during the early adolescence decreased the delta and increment values of weight and TA volume in male but not female SD rats

A group of female and male rats was trained in a forced running wheel twice a day [at ZT14/AM and ZT20/PM during the early adolescence (P27–44)]. The habituation (P26–34) and training (P35–44) phase consisted of a load that increased daily (Fig. 1). Control and experimental male and female rats showed similar weights before the training (P28, Fig. 6A). However, only male trained rats showed lower weight delta and increment values than their control (P44, Fig. 6A–A”). Weight differences between control and experimental male rats were observed the 3 last days of the training (Fig. 6A”’), but the weight increment shows these differences 8 days before the end of the training (Fig. 6A””). Control and experimental male, but not female rats, showed similar TA volume before the training (P27, Fig. 6B). Nonetheless, only male trained rats showed lower volume delta and increment values than their control (P45, Fig. 4B–B”).

### AM and PM forced running during the early adolescence decreased AT% delta and increment values in male but not female SD rats

Control and experimental female and male rats showed similar TA AT% before the training (P27, Fig. 7A; Sup. 5; Sup. 6; Vids. 7–14). However, only male trained rats showed lower AT% delta and increment values than their control (P45, Fig. 7A’–A”). After splitting TA SAT% and VAT% contents, we observed that control and experimental male and female rats showed similar SAT% and VAT% before the training (P27, Fig. 7B,C; Sup. 5; Sup. 6). But only male trained rats showed lower SAT% and VAT% delta and increment values than their control (P45, Fig. 7B’,B”,C’,C”). The SAT:VAT ratio was higher in experimental males, but not females, than their control after the training (Fig. 7D). Regarding the SAT% versus VAT%, only experimental males showed lower VAT% than SAT% after the training program (P45; Fig. 7E–F’). The TA LEAN% delta and increment values were higher in both female and male experimental groups on P45 (Fig. 7G’–G”). Finally, the TA BONE% delta and increment values were lower in experimental females, but higher in experimental males on P45 (Fig. 7H’–H”).

## Discussion

In the present research, we defined a baseline to determine the tendency of weight, body volume, and relative adipose, lean and bone contents of female and male Sprague–Dawley rats during two life stages: adolescence (P30–60) and early adulthood (P60–90). Our results highlight that, during adolescence, both female and male SD rats showed the highest increment in weight, body volume, and AT%, but also the highest decrease of BONE%. In our baseline data, males showed higher weight, body volume, SAT% and delta LEAN%, but lower delta BONE% than females. Sex-dependent differences during the adolescence were also observed in the maturation of the prefrontal cortex of Long-Evans rats^57^.

The literature assessing the body composition before, during and after adolescence in female and male rodents by in vivo techniques (e.g. MRI, CT) is scarce. Studies determining the body composition in rats, were mainly developed in young or adult periods^32,49,58,59^. The relative body fat (or adipose tissue) of SD rats determined by Schoeffner et al.^33^, with an ex-vivo technique, was lower than in our study, and did not seem to increase during the adolescence and young adulthood. These differences might be explained by the gravimetric method used to determine the lipid content, as well as the data acquisition (grams) and its normalization method^33^. In addition, ex-vivo techniques imply the utilization of cohorts, which might induce bias due to the litter differences. CT scan is appropriate to evaluate longitudinal changes in body composition, assuming that the exposure to radiation during our study is not in range to produce tissue damages or morphological alterations in rats^60,61^. Nonetheless our CT images and data, normalized with the volume, indicate there are drastic changes in the relative adipose content during adolescence. Tekus et al.^49^ proposed that the body composition of the region between L1-3 can be used to estimate the whole-body composition. Nonetheless, in our study, we observed some differences between the body composition in the TA region and the OBA region, especially regarding relative lean and bone contents (Fig. 5A–F”; Sup. 3A–F”). Thus, we suggest that estimating the body composition from relatively lesser portions of the body could result in errors, since further studies are required to understand how body composition is regulated in different body regions and organs. On the other hand, many works reported their body composition data as absolute values or relative to body weight^29,30,58^. Some of these studies also include delta body weight^30^ or the percentual increment^59,62^. In order to obtain the delta and incremental values, it is necessary to perform pre and post data acquisition. In our data, the absolute value (e.g. weight) or relative content (e.g. adipose tissue) highlighted the general trend of the values throughout the experiment. On the other hand, the delta value was used to determine the size of the change between two points in time (e.g. females vs. males during adolescence). However, the percentual increment better reflected the fold change from the initial to the final time points, and therefore was better for the comparison of two periods (e.g. adolescence vs. young adulthood), although it also detected the differences between conditions (e.g. experimental vs. control). As a result, the delta or the increment values were not used interchangeably. As an example the female delta VAT% was similar in adolescence and the adulthood, but the increment revealed the VAT% increased more in the adolescence (Fig. 4H’,H”,I’,I”).

We found that a forced wheel training of 18 days during the early adolescence of rats has sex-dependent effects. AM and PM forced running during the early adolescence decreased weight, body volume and relative adipose delta and increment values in males only. The sex-dependent effects in -at least- the body weight could be partially explained by experiments showing that pre-adolescent gonadectomy in rats reduced the sex-dependent differences in body weight and prefrontal cortex maturation^63,64^. In our results, males started with higher SAT% than VAT%, and exercise prevented both SAT% and VAT% gain compared to the control, with a higher effect on VAT% (Fig. 7E–F’). These results suggest that our exercise program has increased benefits in males’ VAT, and therefore can potentially reduce the risk of developing pathologies related to visceral adiposity^65–68^, and preventing critical COVID-19 illness^69^. The absence of effect in the adipose delta values of female rats might suggest that the prevalence of obesity during adolescence in humans is greater in females than males. However, epidemiological studies based on body mass index suggest there is no more prevalence of overweight and obesity in girls than boys^70^. Further studies are required to understand if a different exercise load is required to decrease the adipose content of adolescent female rats. The decrease of female SAT% observed in control rats between P27 and P45 contrasts with the observed increase between P30 and P60 of the baseline rats, suggesting that SAT% in females increases mainly after P45. Regarding the lean tissue, in our study both trained females and males showed higher delta lean values. On the other hand, trained females showed lower bone gains than their control, while trained males showed higher bone gain. These results are in line with the available evidence in adolescent humans showing that exercise enhances lean body mass^71–73^.

Some research results suggest the sex-dependent effects of exercise on body composition might vary depending on the life stage and the voluntary or forced condition of the exercise^25,58,59^. Cortright et al.^25^ observed sex-dependent effects on the weight and body composition of male and female SD rats after 63 days of voluntary running from early adolescence through young adulthood (P21–84). However, only after 49 days of voluntary running the effects in weight were observed (P70, young adulthood). In Cortright’s study, the body composition was determined ex-vivo on P84, and therefore the delta and percentual increment values were not obtained, but no effect was observed in females. Nonetheless, in our study we found that forced wheel running produced body weight differences in adolescent males even 10 days after exercise onset, and the body composition differences were observed in-vivo after 18 days of forced running. Complementarily, the study of Applegate et al.^58^ found that after a training in forced treadmill during young adulthood (P78-100) of Osborne-Mendel rats, only males decreased in weight. In the same study, both males and females showed lower body fat after the exercise program, but the effect was higher in males than females. Finally, a voluntary running for 12 months in adult mice (1-year-old) decreased body weight, body fat and lean contents in both females and males^59^. Taken together, these data suggest that the sex-dependent results we observed with forced wheel exercise may occur mainly during the adolescence.

Changes in body composition observed after the training program (P27–P45) in our study occur when testosterone in male and estradiol in female are beginning to increase. Serum testosterone levels in SD rats are close to zero during early adolescence, with greater increase from P40 to P44. Serum estradiol levels in female SD rats are detectable during adolescence and increases gradually, reaching adult values at P48^74^. An acute increase of testosterone and growth hormone serum concentrations can be observed after a resistance exercise^75^. Further studies will need to determine if these hormonal increases occur in adolescent rats during the aerobic training and if these hormonal changes can be causally related with the changes in body composition. Also, pre-adolescent gonadectomy studies could help to clarify possible roles of testosterone and estradiol in the observed differences in body composition^63,64^.

Male decrease in body weight and fat composition, observed after our training program during early adolescence, should be analyzed in relation to neural circuits that sense energy expenditure and thermogenesis. Hypothalamic nuclei such as arcuate (through POMC and AGRP neurons) and dorsomedian, involved in the control of food intake and energy expenditure^76^ are affected by the adipokine leptin, secreted by the adipose tissue. This hormone reduces food intake and increases energy expenditure, it shows higher sensitivity in females than males and was observed that exercise can decrease its concentrations in woman^77,78^. Sexual dimorphism was determined in diet-induced rodent obesity models, with females being more resistant to storing fat^79^. Also, a better improvement in food intake and weight was observed in some obesity treatments in female mice, representing a sexual dimorphism probably dependent of leptin effects^80^.

In summary, our data indicate that the most drastic changes in the body composition of SD rats occur during the adolescence when compared with the young adulthood, and that adolescent male rats showed higher susceptibility to body weight and body composition changes than females when subjected to forced wheel exercise. Also, this susceptibility seems to occur mainly in the adolescence. Future studies should analyze the molecular mechanisms which mediate the decrease of adipose tissue under physical activity in males but not females during the early adolescence.

## Supplementary Information

Supplementary Figures.

Supplementary Video 1.

Supplementary Video 2.

Supplementary Video 3.

Supplementary Video 4.

Supplementary Video 5.

Supplementary Video 6.

Supplementary Video 7.

Supplementary Video 8.

Supplementary Video 9.

Supplementary Video 10.

Supplementary Video 11.

Supplementary Video 12.

Supplementary Video 13.

Supplementary Video 14.

## Data Availability

All the data and protocols of the procedures are available on reasonable request.

## References

[1] Caballero A, Granberg R, Tseng KY (2016). Mechanisms contributing to prefrontal cortex maturation during adolescence. Neurosci. Biobehav. Rev..

[2] Caballero A, Tseng KY (2016). GABAergic function as a limiting factor for prefrontal maturation during adolescence. Trends Neurosci..

[3] Gugusheff JR, Ong ZY, Muhlhausler BS (2015). The early origins of food preferences: targeting the critical windows of development. FASEB J..

[4] Selemon LD (2013). A role for synaptic plasticity in the adolescent development of executive function. Transl. Psychiatry.

[5] Spear LP (2000). The adolescent brain and age-related behavioral manifestations. Neurosci. Biobehav. Rev..

[6] Hills AP, Andersen LB, Byrne NM (2011). Physical activity and obesity in children. Br. J. Sports Med..

[7] Nader PR (2008). Moderate-to-vigorous physical activity from ages 9 to 15 years. JAMA.

[8] Siervogel RM, Demerath EW, Schubert C, Remsberg KE, Chumlea WC, Sun S (2003). Puberty and body composition. Horm. Res. Paediatr..

[9] Boreham C, Robson PJ, Gallagher AM, Cran GW, Savage JM, Murray LJ (2004). Tracking of physical activity, fitness, body composition and diet from adolescence to young adulthood: the Young Hearts Project, Northern Ireland. Int. J. Behav. Nutr. Phys. Act..

[10] Petridou A, Siopi A, Mougios V (2019). Exercise in the management of obesity. Metabolism.

[11] Buie JJ, Watson LS, Smith CJ, Sims-Robinson C (2019). Obesity-related cognitive impairment: the role of endothelial dysfunction. Neurobiol. Dis..

[12] da Rocha GL, Crisp AH, de Oliveira MR, da Silva CA, Silva JO, Duarte AC (2016). Effect of high intensity interval and continuous swimming training on body mass adiposity level and serum parameters in high-fat diet fed rats. Sci. World J..

[13] Folgueira C, Beiroa D, Porteiro B, Duquenne M, Puighermanal E, Fondevila MF (2019). Hypothalamic dopamine signaling regulates brown fat thermogenesis. Nat. Metab..

[14] Ghaedi H, Faramarzi M, Samani KG, Banitalebi E, Jazi AA (2019). The effect of different endurance exercise intensities on the expression of RIP140 protein in visceral adipose tissue in diabetic rats. Iran. J. Diabetes Obes..

[15] Graham LC, Grabowska WA, Chun Y, Risacher SL, Philip VM, Saykin AJ (2019). Exercise prevents obesity-induced cognitive decline and white matter damage in mice. Neurobiol. Aging.

[16] Guo X, Tao X, Tong Q, Li T, Dong D, Zhang B (2019). Impaired AMPKCGRP signaling in the central nervous system contributes to enhanced neuropathic pain in highfat dietinduced obese rats, with or without nerve injury. Mol. Med. Rep..

[17] Lambert GW, Schlaich MP, Eikelis N, Lambert EA (2019). Sympathetic activity in obesity: a brief review of methods and supportive data. Ann. N. Y. Acad. Sci..

[18] Negaresh R, Motl RW, Zimmer P, Mokhtarzade M, Baker JS (2019). Effects of exercise training on multiple sclerosis biomarkers of central nervous system and disease status: a systematic review of intervention studies. Eur. J. Neurol..

[19] Saxton SN, Withers SB, Heagerty AM (2019). Emerging roles of sympathetic nerves and inflammation in perivascular adipose tissue. Cardiovasc. Drugs Ther..

[20] Shirvani H, Arabzadeh E (2020). Metabolic cross-talk between skeletal muscle and adipose tissue in high-intensity interval training vs. moderate-intensity continuous training by regulation of PGC-1alpha. Eat Weight Disord..

[21] Silva VRR, Lenhare L, Katashima CK, Morari J, Assis MA, Gaspar RS (2019). TGF-beta1 downregulation in the hypothalamus of obese mice through acute exercise. J. Cell. Biochem..

[22] Tsai CL, Pan CY, Chen FC, Huang TH, Tsai MC, Chuang CY (2019). Differences in neurocognitive performance and metabolic and inflammatory indices in male adults with obesity as a function of regular exercise. Exp. Physiol..

[23] Uysal N, Kiray M, Sisman A, Camsari U, Gencoglu C, Baykara B (2015). Effects of voluntary and involuntary exercise on cognitive functions, and VEGF and BDNF levels in adolescent rats. Biotech. Histochem..

[24] Coqueiro AY, Raizel R, Hypólito TM, Tirapegui J (2017). Effects of supplementation with L-glutamine and L-alanine in the body composition of rats submitted to resistance exercise. Rev. Bras. Ciênc. Esporte.

[25] Cortright RN, Chandler MP, Lemon PWR, Dicarlo SE (1997). Daily exercise reduces fat, protein and body mass in male but not female rats. Physiol. Behav..

[26] Crews EL, Fuge KW, Oscai LB, Holloszy JO, Shank RE (1969). Weight, food intake, and body composition: effects of exercise and of protein deficiency. Am. J. Physiol. Leg. Content.

[27] Narath E, Skalicky M, Viidik A (2001). Voluntary and forced exercise influence the survival and body composition of ageing male rats differently. Exp. Gerontol..

[28] Shinoda M, Latour M, Lavoie JM (2002). Effects of physical training on body composition and organ weights in ovariectomized and hyperestrogenic rats. Int. J. Obes..

[29] Bloomer R, Schriefer J, Gunnels T, Lee S-R, Sable H, Van Der Merwe M (2018). Nutrient intake and physical exercise significantly impact physical performance, body composition, blood lipids, oxidative stress, and inflammation in male rats. Nutrients.

[30] Caton SJ, Bielohuby M, Bai Y, Spangler LJ, Burget L, Pfluger P (2012). Low-carbohydrate high-fat diets in combination with daily exercise in rats: effects on body weight regulation, body composition and exercise capacity. Physiol. Behav..

[31] Emery PW, Rothwell NJ, Stock MJ, Winter PD (1984). Chronic effects of β2 agonists on body composition and protein synthesis in the rat. Biosci. Rep..

[32] Pitts GC (1984). Body composition in the rat: interactions of exercise, age, sex, and diet. Am. J. Physiol. Regul. Integr. Comp. Physiol..

[33] Schoeffner DJ (1999). Organ weights and fat volume in rats as a function of strain and age. J. Toxicol. Environ. Health A.

[34] Hall CB (1989). Estimation of rat body composition using tetrapolar bioelectrical impedance analysis. Nutr. Rep. Int..

[35] Lesser GT, Deutsch S, Markofsky J (1973). Aging in the rat: longitudinal and cross-sectional studies of body composition. Am. J. Physiol. Leg. Content.

[36] Rothwell NJ, Stock MJ (1979). Regulation of energy balance in two models of reversible obesity in the rat. J. Comp. Physiol. Psychol..

[37] Goldberg EK, Fung EB (2020). Precision of the hologic DXA in the assessment of visceral adipose tissue. J. Clin. Densitom..

[38] Chen YI, Cypess AM, Sass CA, Brownell AL, Jokivarsi KT, Kahn CR (2012). Anatomical and functional assessment of brown adipose tissue by magnetic resonance imaging. Obesity.

[39] Moreno-Fernandez J, Diaz-Castro J, Alferez MJM, Lopez-Aliaga I (2019). Iron deficiency and neuroendocrine regulators of basal metabolism, body composition and energy expenditure in rats. Nutrients.

[40] Romu T, Elander L, Leinhard OD, Lidell ME, Betz MJ, Persson A (2015). Characterization of brown adipose tissue by water-fat separated magnetic resonance imaging. J. Magn. Reson. Imaging..

[41] Baba S, Jacene HA, Engles JM, Honda H, Wahl RL (2010). CT Hounsfield units of brown adipose tissue increase with activation: preclinical and clinical studies. J. Nucl. Med..

[42] Wang X, Minze LJ, Shi ZZ (2012). Functional imaging of brown fat in mice with 18F-FDG micro-PET/CT. J. Vis. Exp..

[43] Beaucage KL, Pollmann SI, Sims SM, Dixon SJ, Holdsworth DW (2016). Quantitative in vivo micro-computed tomography for assessment of age-dependent changes in murine whole-body composition. Bone Rep..

[44] Granton PV, Norley CJ, Umoh J, Turley EA, Frier BC, Noble EG (2010). Rapid in vivo whole body composition of rats using cone beam muCT. J. Appl. Physiol. (1985).

[45] Judex S, Luu YK, Ozcivici E, Adler B, Lublinsky S, Rubin CT (2010). Quantification of adiposity in small rodents using micro-CT. Methods.

[46] Luu YK, Lublinsky S, Ozcivici E, Capilla E, Pessin JE, Rubin CT (2009). In vivo quantification of subcutaneous and visceral adiposity by micro-computed tomography in a small animal model. Med. Eng. Phys..

[47] Metzinger MN, Miramontes B, Zhou P, Liu Y, Chapman S, Sun L (2014). Correlation of X-ray computed tomography with quantitative nuclear magnetic resonance methods for pre-clinical measurement of adipose and lean tissues in living mice. Sensors (Basel).

[48] Chusyd DE, Wang D, Huffman DM, Nagy TR (2016). Relationships between rodent white adipose fat pads and human white adipose fat depots. Front. Nutr..

[49] Tekus E, Miko A, Furedi N, Rostas I, Tenk J, Kiss T (2017). Body fat of rats of different age groups and nutritional states: assessment by micro-CT and skinfold thickness. J. Appl. Physiol..

[50] Clark DP, Badea CT (2014). Micro-CT of rodents: state-of-the-art and future perspectives. Phys. Med..

[51] Marzola P, Boschi F, Moneta F, Sbarbati A, Zancanaro C (2016). Preclinical in vivo imaging for fat tissue identification, quantification, and functional characterization. Front. Pharmacol..

[52] Toval A, Banos R, De la Cruz E, Morales-Delgado N, Pallares JG, Ayad A (2017). Habituation training improves locomotor performance in a forced running wheel system in rats. Front. Behav. Neurosci..

[53] Toval A, Garrigos D, Kutsenko Y, Popović M, Do-Couto BR, Morales-Delgado N (2021). Dopaminergic modulation of forced running performance in adolescent rats: role of striatal D1 and extra-striatal D2 dopamine receptors. Mol. Neurobiol..

[54] Toval A, Vicente-Conesa F, Martínez-Ortega P, Kutsenko Y, Morales-Delgado N, Garrigos D (2020). Hypothalamic Crh/Avp, plasmatic glucose and lactate remain unchanged during habituation to forced exercise. Front. Physiol..

[55] Percie Du Sert N, Hurst V, Ahluwalia A, Alam S, Avey MT, Baker M (2020). The ARRIVE guidelines 2.0: updated guidelines for reporting animal research. PLOS Biol..

[56] Aubrey J, Esfandiari N, Baracos VE, Buteau FA, Frenette J, Putman CT (2014). Measurement of skeletal muscle radiation attenuation and basis of its biological variation. Acta Physiol. (Oxf.).

[57] Willing J, Juraska JM (2015). The timing of neuronal loss across adolescence in the medial prefrontal cortex of male and female rats. Neuroscience.

[58] Applegate EA, Upton DE, Stern JS (1982). Food intake, body composition and blood lipids following treadmill exercise in male and female rats. Physiol. Behav..

[59] McMullan RC, Kelly SA, Hua K, Buckley BK, Faber JE, Pardo-Manuel De Villena F (2016). Long-term exercise in mice has sex-dependent benefits on body composition and metabolism during aging. Physiol. Rep..

[60] Klinck RJ, Campbell GM, Boyd SK (2008). Radiation effects on bone architecture in mice and rats resulting from in vivo micro-computed tomography scanning. Med. Eng. Phys..

[61] Mustafy T, Benoit A, Londono I, Moldovan F, Villemure I (2018). Can repeated in vivo micro-CT irradiation during adolescence alter bone microstructure, histomorphometry and longitudinal growth in a rodent model?. PLoS ONE.

[62] Friemel CM (2010). SRaSM. Reward sensitivity for a palatable food reward peaks during pubertal developmental in rats. Front. Behav. Neurosci..

[63] Koss WA, Lloyd MM, Sadowski RN, Wise LM, Juraska JM (2015). Gonadectomy before puberty increases the number of neurons and glia in the medial prefrontal cortex of female, but not male, rats. Dev. Psychobiol..

[64] Vetter-O’Hagen CS, Spear LP (2011). The effects of gonadectomy on age- and sex-typical patterns of ethanol consumption in Sprague–Dawley rats. Alcohol. Clin. Exp. Res..

[65] Pappas LE, Nagy TR (2019). The translation of age-related body composition findings from rodents to humans. Eur. J. Clin. Nutr..

[66] Ejtahed H-S, Kelishadi R, Hasani-Ranjbar S, Angoorani P, Motlagh ME, Shafiee G (2019). Discriminatory ability of visceral adiposity index as an indicator for modeling cardio-metabolic risk factors in pediatric population: the CASPIAN-V study. J. Cardiovasc. Thorac. Res..

[67] Motamed N, Khonsari MR, Rabiee B, Ajdarkosh H, Hemasi GR, Sohrabi MR (2017). Discriminatory ability of visceral adiposity index (VAI) in diagnosis of metabolic syndrome: a population based study. Exp. Clin. Endocrinol. Diabetes.

[68] Nusrianto R, Ayundini G, Kristanti M, Astrella C, Amalina N, Muhadi (2019). Visceral adiposity index and lipid accumulation product as a predictor of type 2 diabetes mellitus: The Bogor cohort study of non-communicable diseases risk factors. Diabetes Res. Clin. Pract..

[69] Battisti S, Pedone C, Napoli N, Russo E, Agnoletti V, Nigra SG (2020). Computed tomography highlights increased visceral adiposity associated with critical illness in COVID-19. Diabetes Care.

[70] Ogden CL, Carroll MD, Lawman HG, Fryar CD, Kruszon-Moran D, Kit BK (2016). Trends in obesity prevalence among children and adolescents in the United States, 1988–1994 through 2013–2014. JAMA.

[71] Baxter-Jones ADG, Eisenmann JC, Mirwald RL, Faulkner RA, Bailey DA (2008). The influence of physical activity on lean mass accrual during adolescence: a longitudinal analysis. J. Appl. Physiol..

[72] Larsen MN, Nielsen CM, Ørntoft CØ, Randers MB, Manniche V, Hansen L (2017). Physical fitness and body composition in 8–10-year-old danish children are associated with sports club participation. J. Strength Cond. Res..

[73] Schumann M, Küüsmaa M, Newton RU, Sirparanta A-I, Syväoja H, Häkkinen A (2014). Fitness and lean mass increases during combined training independent of loading order. Med. Sci. Sports Exerc..

[74] Vetter-O’Hagen CS, Spear LP (2012). Hormonal and physical markers of puberty and their relationship to adolescent-typical novelty-directed behavior. Dev. Psychobiol..

[75] West DW, Kujbida GW, Moore DR, Atherton P, Burd NA, Padzik JP, De Lisio M (2009). Resistance exercise-induced increases in putative anabolic hormones do not enhance muscle protein synthesis or intracellular signalling in young men. J. Physiol..

[76] Trotta M, Bello EP, Alsina R, Tavella MB, Ferrán JL, Rubinstein M (2020). Hypothalamic Pomc expression restricted to GABAergic neurons suppresses Npy overexpression and restores food intake in obese mice. Mol. Metab..

[77] Clegg DJ, Riedy CA, Smith KAB, Benoit SC, Woods SC (2003). Differential sensitivity to central leptin and insulin in male and female rats. Diabetes.

[78] Kraemer RR, Chu H, Castracane VD (2002). Leptin and exercise. Exp. Biol. Med..

[79] Casimiro I, Stull ND, Tersey SA, Mirmira RG (2021). Phenotypic sexual dimorphism in response to dietary fat manipulation in C57BL/6J mice. J. Diabetes Complicat..

[80] Bumaschny VF, Yamashita M, Casas-Cordero R, Otero-Corchón V, de Souza FSJ, Rubinstein M, Low MJ (2012). Obesity-programmed mice are rescued by early genetic intervention. J. Clin. Invest..

