# Supplementary material for: Sex-dependent effects of forced exercise in the body composition of adolescent rats
Source: Sci Rep. 2021 May 12;11:10154. doi: 10.1038/s41598-021-89584-8 (PMC8115159; doi:10.1038/s41598-021-89584-8)
Supplement: Supplementary file 1 — Supplementary Figures. [file 41598_2021_89584_MOESM1_ESM.docx]

Supplementary figures for:

**Sex-dependent effects of forced exercise in the body composition of adolescent rats.**

Y. Kutsenko^1,2^, A. Barreda^1,2^, A. Toval^1,2^, D. Garrigos^1,2^, M. Martínez-Morga^1,2^, B. Ribeiro Do-Couto^2,3^, J. L. Ferran^1,2*^.

^1.^ Department of Human Anatomy and Psychobiology, Faculty of Medicine, University of Murcia, Murcia, Spain.

^2.^ Institute of Biomedical Research of Murcia – IMIB, Virgen de la Arrixaca University Hospital.

^3^ Faculty of Psychology, University of Murcia, Murcia, Spain.

* Correspondence: José Luis Ferran [jlferran@um.es](mailto:jlferran@um.es)

**
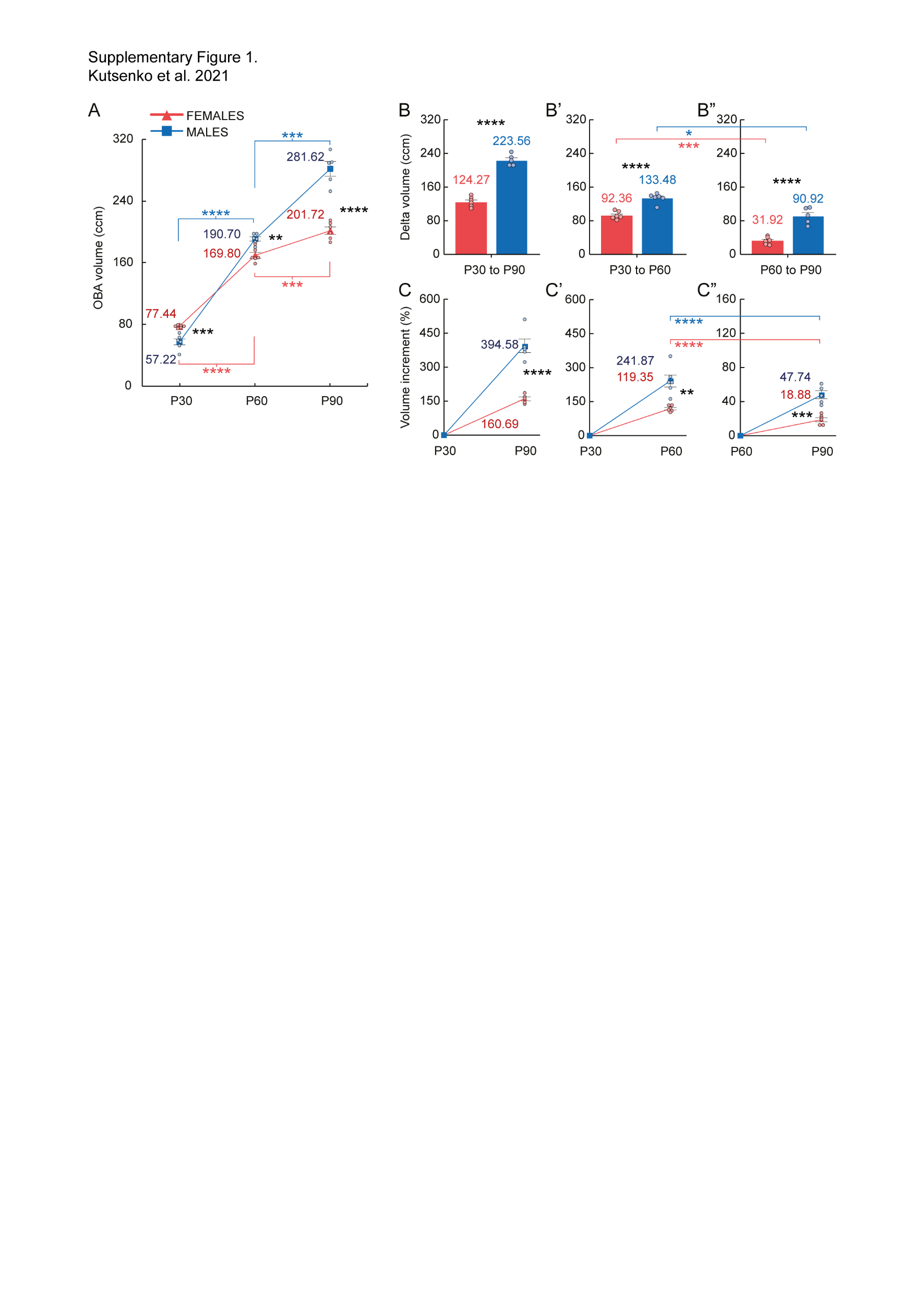
**

**Sup. 1. OBA region volume of female (red) and male (blue) rats.** **A)** OBA volume (ccm). The split-plot ANOVA revealed an interaction between age and sex in OBA volume (F_2,20_ = 102.03, p < .01), with main effects of age (F_2,20_ = 1259.75, p < .01) and sex (F_1,10_ = 32.83, p < .01). **B, B’ and B”)** Delta OBA volume (ccm). **C, C’ and C”)** Percentual increment of OBA volume (%). **Statistics**: Between-subjects effects were followed with Fisher’s LSD post-hoc. Paired T-test was used for within-subjects comparisons. Unpaired T-test was used in delta and increment between-subjects comparisons. Black stars (*): female vs male. Blue symbols: within-male comparisons. Red symbols: within-female comparisons. Values represented as mean and SEM. Significance levels: * p < .05, ** p < .01, *** p < .001, **** p < .0001, ns: p > .05.


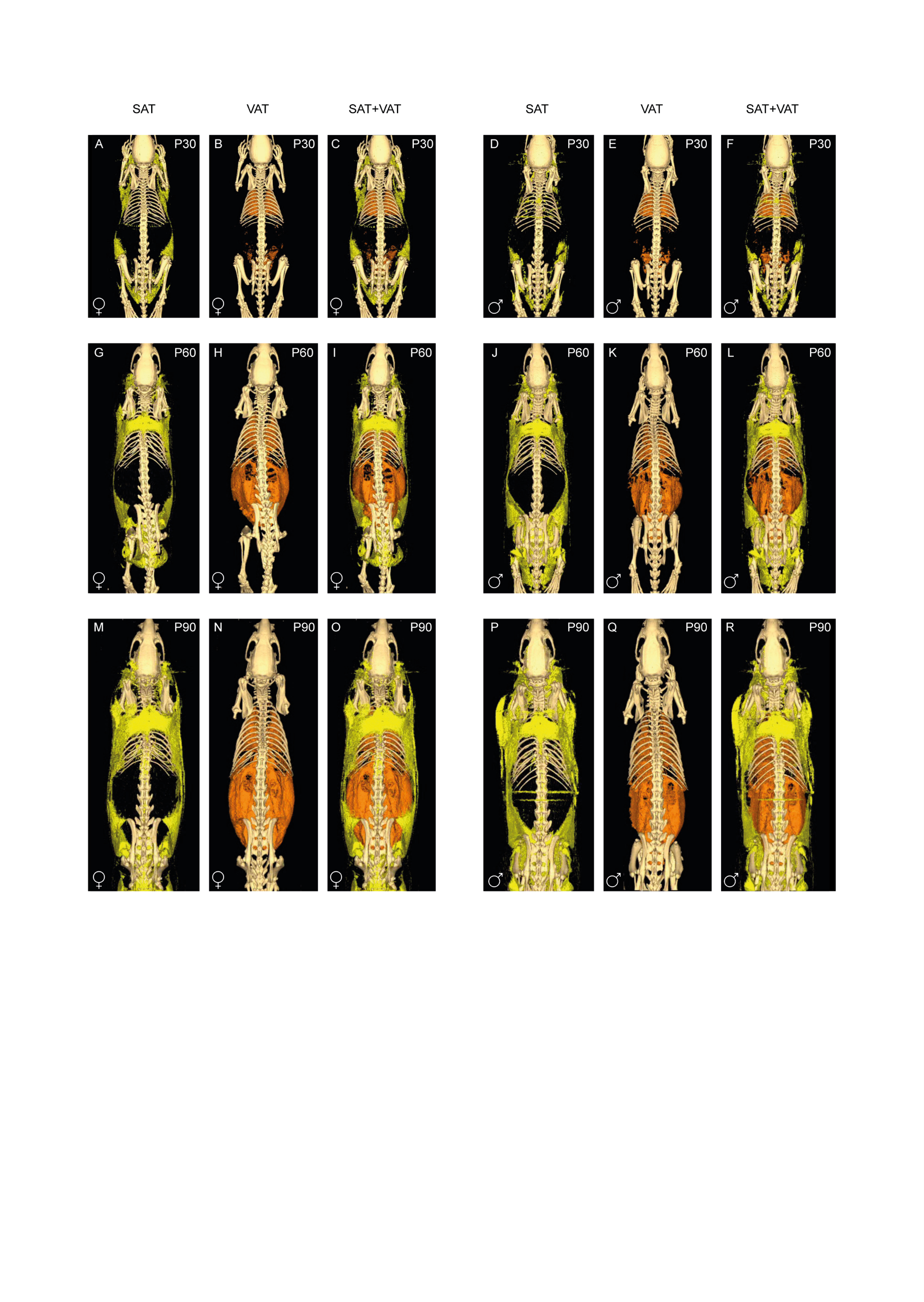


**Sup. 2. Reconstruction of tomography images in 3D of the female and male baseline rats. A-O)** subcutaneous (SAT, yellow), visceral (VAT, orange), and total (SAT and VAT) adipose contents of female rats. **D-R)** SAT, VAT and SAT+VAT contents of male rats.


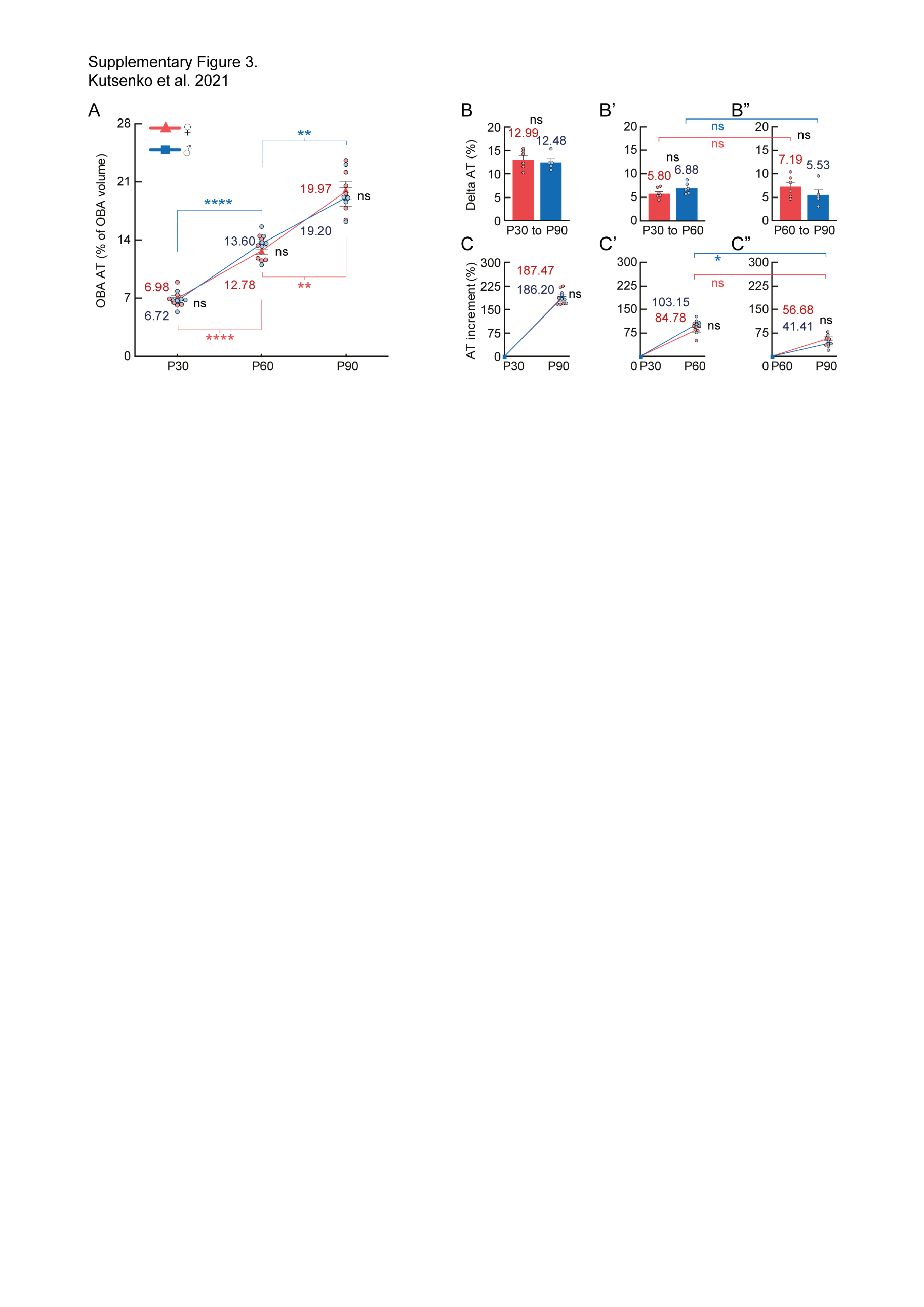


**Sup. 3. Female (red) and male (blue) comparisons of OBA adipose contents. A)** Relative adipose contents (AT%). The split-plot ANOVA revealed a main effect of age in OBA AT% (F_2,20_ = 311.79, p < .01) **B, B’ and B”)** Delta AT%. **C, C’ and C”)** Percentual increment (%) of AT%. **Statistics**: Between-subjects effects were followed with Fisher’s LSD post-hoc. Paired T-test was used for within-subjects comparisons. Unpaired T-test was used in delta and increment between-subjects comparisons. Black stars (*): female vs male. Blue symbols: within-male comparisons. Red symbols: within-female comparisons. Values represented as mean and SEM. Significance levels: * p < .05, ** p < .01, *** p < .001, **** p < .0001, ns: p > .05.


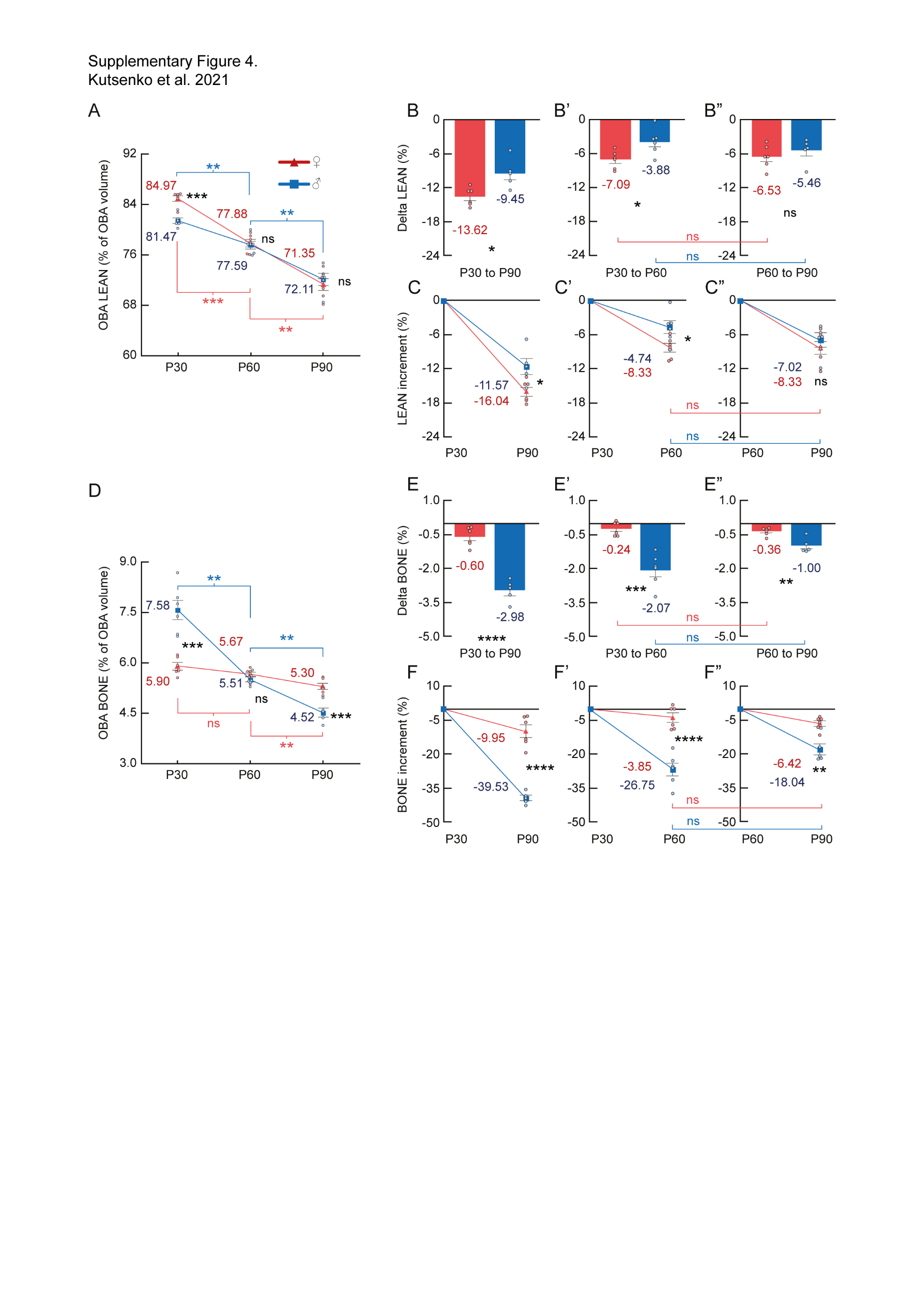


**Sup. 4. Female (red) and male (blue) comparisons of OBA LEAN% and OBA BONE%.** **A)** Relative lean contents (LEAN%). The split-plot ANOVA revealed an interaction between age and sex in OBA LEAN% (F_2,20_ = 7.34, p < .01), with a main effect of age (F_2,20_ = 195.38, p < .01). **D)** Relative bone contents (BONE%). The split-plot ANOVA revealed an interaction between age and sex in OBA BONE% (F_1.5,14.9_ = 50.76, p < .01), with a main effect of age (F_1.5,14.9_ = 106.29, p < .01). **B-B” and E-E”)** Delta LEAN% and delta BONE% (respectively). **C-C” and** **F-F”)** LEAN% and BONE% (respectively) percentual increment (%). **Statistics**: Between-subjects effects were followed with Fisher’s LSD post-hoc. Paired T-test was used for within-subjects comparisons. Unpaired T-test was used in delta and increment between-subjects comparisons. Black stars (*): female vs male. Blue symbols: within-male comparisons. Red symbols: within-female comparisons. Values represented as mean and SEM. Significance levels: * p < .05, ** p < .01, *** p < .001, **** p < .0001, ns: p > .05.


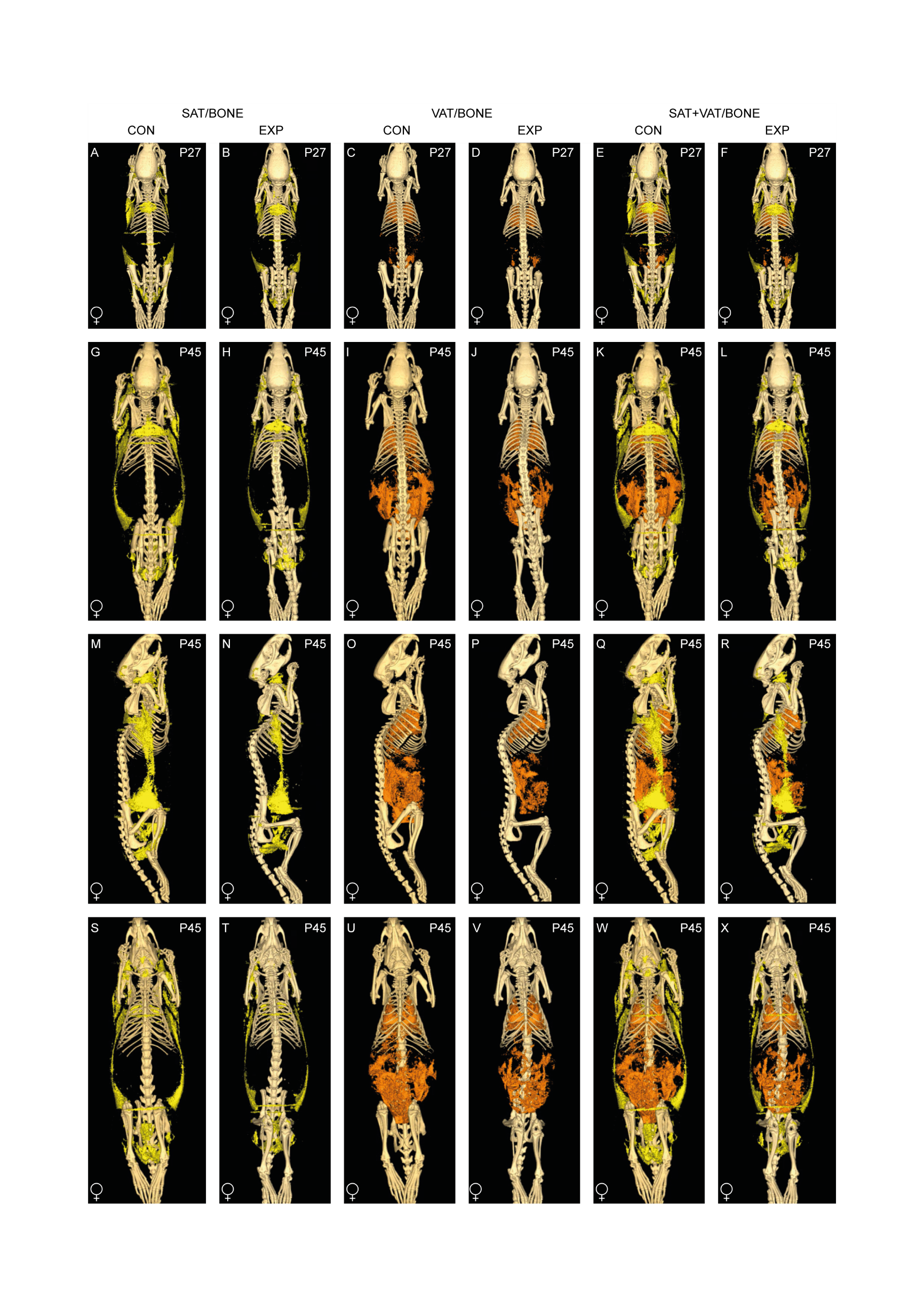


**Sup. 5. Reconstruction of tomography images in 3D of the female control and experimental rats. A-X)** Subcutaneous (SAT, yellow), visceral (VAT, orange), and total (SAT and VAT) adipose tissue contents of control and experimental female rats.


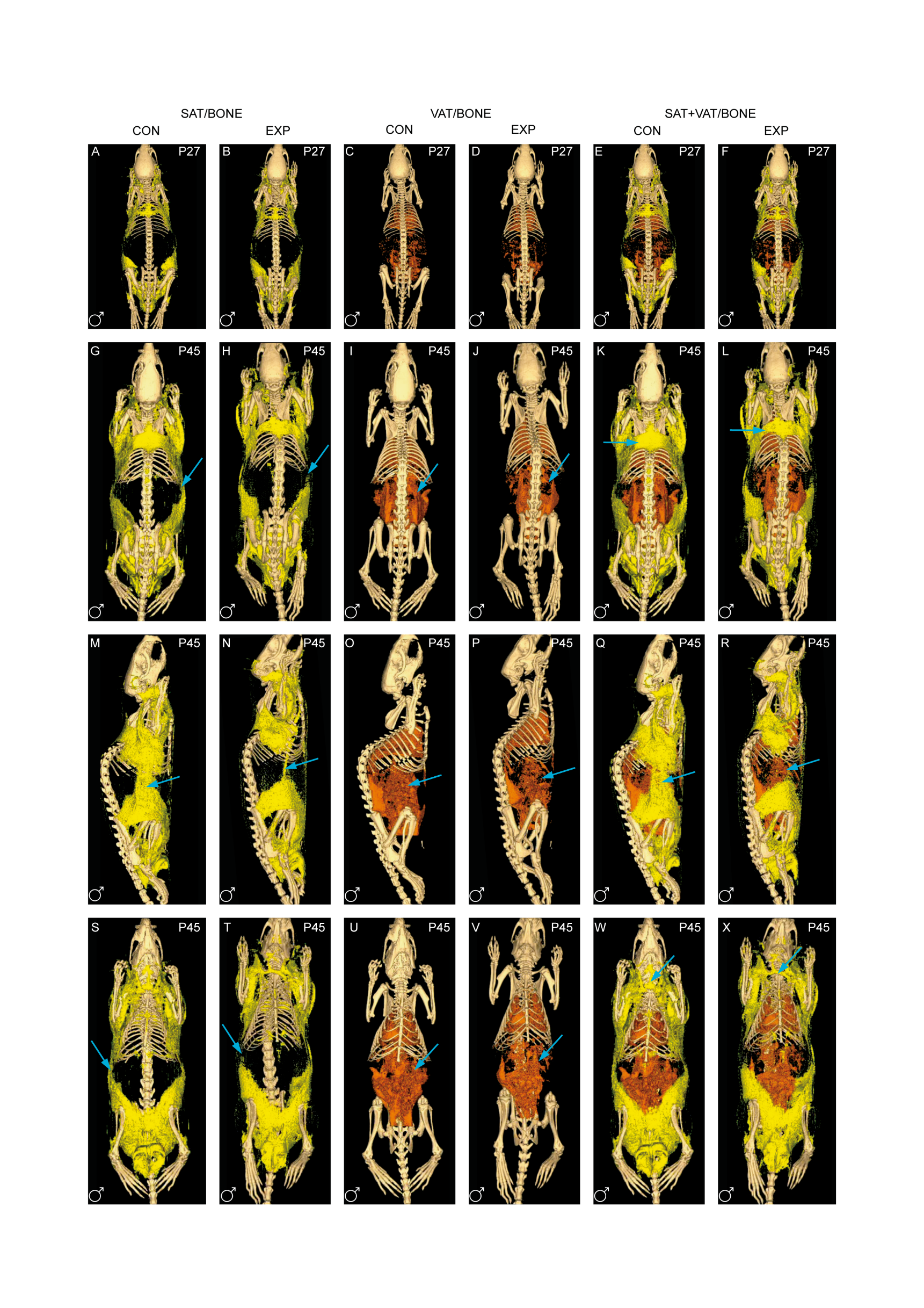


**Sup. 6. Reconstruction of tomography images in 3D of the male control and experimental rats. A-X)** subcutaneous (SAT, yellow), visceral (VAT, orange), and total (SAT and VAT) adipose tissue contents of male rats. Blue arrows highlight visible differences between control and experimental rats.
